# Identification and characterization of *Colletotrichum fioriniae* and *C. fructicola* that cause anthracnose in pecan

**DOI:** 10.3389/fpls.2022.1043750

**Published:** 2022-11-23

**Authors:** Jun Chang, Fengyan Zhai, Yabo Zhang, Di Wang, Jinping Shu, Xiaohua Yao

**Affiliations:** ^1^ Research Institute of Subtropical Forestry, Chinese Academy of Forestry, Fuyang, Hangzhou, Zhejiang, China; ^2^ Henan Institute of Science and Technology Department of Resources & Environment, Xinxiang, Henan, China

**Keywords:** pecan, black-spot diseases, *colletotrichum* spp., pathogen identification, disease resistance

## Abstract

Pecan (*Carya illinoinensis* Wang. K. Koch) is a deciduous tree of the Juglandaceae family with important economic value worldwide. Anthracnose of the pecan leaves and shuck is a devastating disease faced by pecan-growing areas in China. However, the causal species occurring on pecan remain largely unidentified. we collected samples of diseased pecan from the provinces of China, Leaves and fruits affected by anthracnose were sampled and subjected to fungus isolation, The morphological characters of all strains were observed and compared; Multi-locus phylogenetic analyses [Internally transcribed spacer (ITS), Actin (ACT), Calmodulin (CAL), Chitin synthase (CHS1), Glyceraldehyde 3-phosphate dehydrogenase (GAPDH), and b-tubulin (TUB2)] were performed on selected representative strains; examine their pathogenicity on leaves of pecan.The results showed that: (1) resulting in a total of 11 *Colletotrichum* isolates, Two *Colletotrichum* species were identifified to be *C. fioriniae* and *C. fructicola*; (2) Pathogenicity tests revealed that both species caused black spots on pecan leaves and fruit, The virulence of the different isolates varied substantially, with *C. fioriniae* PCJD179 being the most virulent; (3) The susceptibility levels of pecan tree varieties, ‘Mahan’ and ‘Kanza’, were determined, No significant differences were observed in the lesion sizes produced by the various isolates in ‘Kanza’, while there were signifificant differences in ‘Mahan’. This study is thefifirst to determine that *C. fructicola* and *C. fioriniaecan* cause anthracnose in pecan in China. It improves the understanding of the species that cause anthracnose in pecan and provides useful information for the effective control of this disease in China.

## Introduction

Pecan (*Carya illinoinensis* Wang. K. Koch) is a deciduous tree of the Juglandaceae family. It is an important economic nut-producing tree worldwide. In China, pecan is planted mostly from 24 to 40° N in 14 provinces, mainly in Anhui, Zhejiang, Jiangsu, and Yunnan (Yao et al., 2014). In 2014, commercial pecan orchards covered ~8,500 ha in China (Zhang et al., 2015). By the end of 2020, the total planting area in the country had grown to approximately 68,000 ha. The national output in 2020 was 2,000 tons, while it was 4,500 tons in 2021 (personal investigation).

*Colletotrichum* is an important pathogenic fungus worldwide. It has numerous species and a wide plant host range. However, studies on its pathogenicity against pecan are limited. Pecan anthracnose caused by *Glomerella cingulata* (stonem.) was first reported in Georgia as early as 1914 (Rand, 1914). Subsequently, three *Colletotrichum* spp. were established to infect pecan: *C. gloeosporioides*, *C. siamense*, and *C. nymphaeae* (Mantz et al., 2010; Poletto et al., 2019; Zhang et al., 2019; Oh et al., 2021).

**Figure 1:**
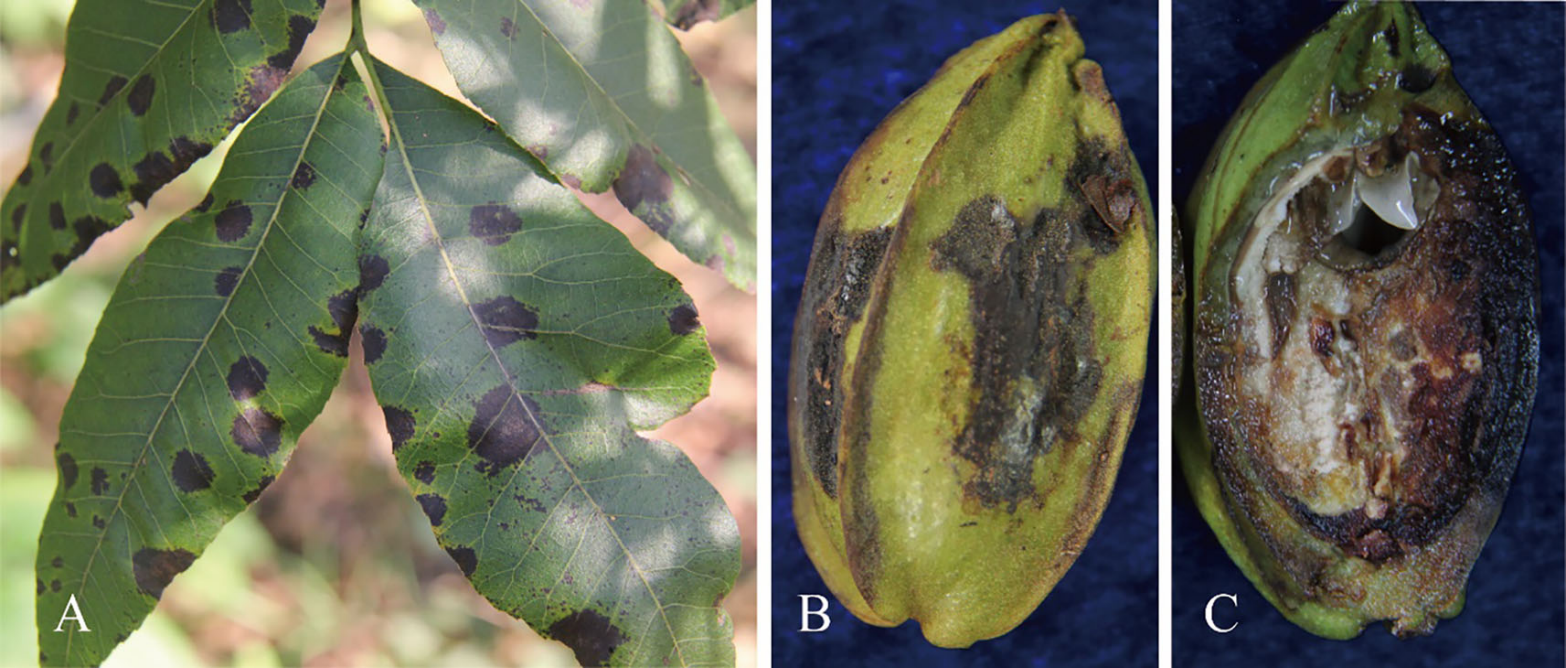
Visible symptoms caused by *Colletotrichum* spp. on pecan. **(A)** Irregular necrotic spots on leaves. **(B)** Irregular grey lesions on the pericarp. **(C)** Internal symptoms in the fruit.

Anthracnose of the leaves and shuck in pecan is among the most devastating diseases that inflict the pecan-growing areas of China. On leaves, the lesions are irregular, necrotic, and usually surrounded by chlorotic rings. They can merge to form large necrotic areas. On the pecan shucks, the lesions are black, irregular, and slightly sunken (**Figures 1A–C**). Light yellow spots appear first, which enlarge and finally develop into irregularly shaped lesions with a chlorotic halo (Zhang et al., 2019). Visible symptoms of shuck include darkening, indentations, and irregularly shaped lesions (Oh et al., 2021). Other symptoms consist of circular lesions of up to 5 mm with clearly delineated or cracked centers, dark brown margins, and a yellowish halo on the leaves (Poletto et al., 2019). Serious infections by such fungi can thus result in autumn defoliation, fruit drop, and a decline in nut quality (Worley, 1979). It is crucial to accurately grasp the control time and select efficient fungicides, which requires the accurate identification of *Colletotrichum* spp. Therefore, the objective of the present study was to characterize and identify *Colletotrichum* isolates obtained from pecan tissues using morphological and molecular tools and to determine their pathogenicity in pecan.

## Materials and methods

### Sampling and fungal isolation

Samples of pecan leaves and shuck displaying typical anthracnose symptoms were collected from pecan trees in Jiande, Zhejiang Province; Ji’an, Jiangxi Province; and Yuxi, Yunnan Province in August 2018 and 2019 (**Table 1**). This period generally covers the early nut maturation and filling stages. Sampling followed the procedures described by Cai et al. (2009) and Prihastuti et al. (2009). Briefly, leaf and fruit surfaces were rinsed with distilled water, disinfected with 75% alcohol for 30 s, disinfected with 3% sodium hypochlorite for 2–3 min, rinsed with sterile water thrice, and dried with sterilized paper. Pieces were sliced from the junctions of diseased and healthy tissues, which were cut into squares with a side length of 3–4 mm. The excised tissues were placed onto potato dextrose agar (PDA, 20% diced potato, 2% glucose, and 1.5% agar in distilled water) plates and incubated at 28°C.Conidia were also collected, suspended in sterilized water, diluted to a concentration of 1 × 10^4^ conidia per mL, and spread onto the surface of water agar (WA, 2% agar in distilled water) to generate discrete colonies (Choi et al., 1999). Six single colonies of each isolate were picked up with a sterilized needle (insect pin, 0.5 mm diameter) and transferred onto PDA plates.

**Table 1:** A list of *Colletotrichum* isolates collected from pecan based on preliminary identification.

Isolate number	Collectionlocations	Latitude (°North)	Longitude (°East)	Host tissue	Sampling times
JD756	Jiande, Zhejiang	29.48	119.28	Shuck	Aug 2018
JD119	Jiande, Zhejiang	29.48	119.28	Shuck	Aug 2018
JD29	Jiande, Zhejiang	29.48	119.28	Shuck	Aug 2018
JD12	Jiande, Zhejiang	29.48	119.28	Shuck	Aug 2018
JD32	Jiande, Zhejiang	29.48	119.28	Shuck	Aug 2018
JD179	Jiande, Zhejiang	29.48	119.28	Shuck	Aug 2018
JD7536	Jiande, Zhejiang	29.48	119.28	Shuck	Aug 2018
JX0731	Ji’an, Jiangxi	27.12	114.98	Shuck	Aug 2019
JX073	Ji’an, Jiangxi	27.12	114.98	Shuck	Aug 2019
YN191	Yuxi, Yunnan	24.35	102.55	Leaf	Aug 2019
YN1751	Yuxi, Yunnan	24.35	102.55	Leaf	Aug 2019

### Morphological characterization

The isolates were purified using single spore isolation and stored at 4°C on PDA slants for further use. They were stored on filter paper at -80°C for long-term preservation. Isolates were transferred from PDA slants to PDA plates and cultivated at 28°C under a 12-h photoperiod for 14 days. The following morphological characteristics were recorded: conidia, appressoria, conidiomata, and conidiophores. The mean lengths and widths of 100 randomly selected conidia from each isolate were measured using 100× magnification in a microscope (Nikon Ti-S inverted microscope, Japan). Among the 36 isolates thus obtained, 11 representative isolates were selected for further multi-locus phylogenetic analyses based on geographical location, morphology (e.g., colony shape and color and other physical characteristics of aerial mycelia and conidia), and ITS sequences.

### DNA extraction

The genomic DNA of each isolate was isolated from 0.5 g of fresh hyphae using a DNA extraction kit (TaKaRa Bioengineering Co., Ltd, Dalian, China) and stored at −20°C. The *ITS*, *GAPDH*, *ACT*, *TUB2*, *CHS*-1, and *CAL* sequences were amplified and sequenced as previously described in Weir et al. (2012). The primers used, along with their respective sequences, are presented in **Table 2**.

**Table 2:** Primers used in this study.

Gene	Product name	Primer	Sequence (5’–3’)	Reference
ITS	*Internal transcribed spacer*	ITS-1	TCCGTAGGTGAACCTGCGC	White et al., 1990
		ITS-4	TCCTCCGCTTATTGATATC	White et al., 1990
ACT	*Actin*	ACT-512F	ATG TGC AAG GCC GGT TTC GC	Carbone and Kohn, 1999
		ACT-783R	TAC GAG TCC TTC TGG CCC AT	Carbone and Kohn, 1999
CAL	*Calmodulin*	CL1C	GAA TTC AAG GAG GCC TTC TC	O’Donnell et al., 2000
		CL2C	CTT CTG CAT GAG CTG GAC	O’Donnell et al., 2000
CHS¬I	*Chitin synthase*	CHS-79F	TGG GGC AAG GAT GCT TGG AAG	Carbone and Kohn, 1999
		CHS-345R	TGG AAG AAC CAT CTG TGA GAG TTG	Carbone and Kohn, 1999
GAPDH	*Glyceraldehyde-3-phosphate dehydrogenase*	GDF	GCC GTC AAC GAC CCC TTC ATT GA	Templeton et al., 1992
		GDR	GGG TGG AGT CGT ACT TGA GCA TGT	Templeton et al., 1992
TUB2	*β-Tubulin*	T1	AAC ATG CGT GAG ATT GTA AGT	O’Donnell and Cigelnik, 1997
		Bt2b	ACC CTC AGT GTA GTG ACC CTT GGC	Glass and Donaldson, 1995

### PCR amplification

The PCR was performed according to the methods of Weir et al. (2012). All the PCR reactions were conducted in 25-μl volumes containing 12.5 μl PCR MasterMix (TIANGEN BIOTECH (BEIJING) CO., LTD., Beijing, China), 10 μM primers (both), 1 μl of the template DNA (20 ng μl^-1^), and 9.5 μl of double-distilled H_2_O. The PCR program to amplify *ACT* and *GAPDH* included a denaturation step at 94°C for 5 min, followed by 35 cycles of 94°C for 45 s, 59°C for 30 s, and 72°C for 2 min. The final cycle comprised 72°C for 10 min. The amplifications of *ITS*, *TUB2*, *CHS*-*1*, and *CAL* were also performed similarly with annealing temperatures of 56, 58, 58, and 57°C, respectively. The amplification products were analyzed on a 1.0% agarose gel in tris/borate/EDTA buffer. The PCR products were then purified and sequenced at Hangzhou Shangya Biotechnology Co., Ltd (Zhejiang, China).

### Phylogenetic analysis

The reference sequences for *ITS*, *GAPDH*, *CHS-1*, *ACT*, *TUB2*, and *CAL* were downloaded from NCBI (**Table 3**). The forward and reverse sequences were edited and assembled using DNAMAN v. 8.0 (Lynnon Biosoft). According to the sequence of ITS-ACT-CAL-CHS-1-GAPDH-TUB2, six loci were combined and aligned using BioEdit v. 7.2.5 (Hall, 1999). Bayesian analyses were performed on concatenated alignments using MrBayes v. 3.2.1 (Ronquist et al., 2012). Maximum likelihood (ML) analyses were performed on the multilocus alignment using IQ-TREE (Nguyen et al., 2015), and the nucleotide substitution models were selected by Model Finder (Kalyaanamoorthy et al., 2017).

**Table 3:** Sequences of *Colletotrichum* spp. isolates used in the phylogenetic analysis.

GenBank No.	Accession No.1	Host/Substrate	Country	GenBank No.					
				ITS	GAPDH	CHS-1	ACT	TUB2	CAL
*C. americae-borealis*	CBS 136232		Germany	KM105224	KM105579	KM105294	KM105434	KM105504	–
*C. boninense*	CBS 123755*	*Crinum asiaticum* var. *sinicum*	Japan	JQ005153	JQ005240	JQ005327	JQ005501	JQ005588	JQ005674
*C. clidemiae*	ICMP 18658*	*Clidemia hirta*	USA, Hawaii	JX010265	JX009989	JX009877	JX009537	JX010438	JX009645
*C. coelogynes*	CBS 132504		Germany	NR_160827	MG600776	MG600836	MG600920	MG600980	–
*C. fioriniae*	S22	*Morus alba*	China	KY986890	KY986896	KY986914	KY986902	MF033884	KY986908
*C. fructicola*	DS-2	*Pyrus bretschneideri*	China	KC410780	KC410783	KC410785	KC410781	KC410782	KC410786
*C. fioriniae*	IMI 324996	*Malus pumila*	USA	JQ948301	JQ948631	JQ948962	JQ949622	JQ949952	–
*C. fioriniae*	CBS 126526	*Primula* sp.*, leaf spots*	Netherlands	JQ948323	JQ948653	JQ948984	JQ949644	JQ949974	–
*C. fioriniae*	CBS 124958	*Pyrus* sp.*, fruit rot*	USA	JQ948306	JQ948636	JQ948967	JQ949627	JQ949957	–
*C. fioriniae*	IMI 504882	*Fragaria × ananassa*	New Zealand	KT153562	KT153552	KT153547	KT153542	KT153567	–
*C. fructicola*	ICMP 18581*, CBS 130416	*Coffea arabica*	Thailand	JX010165	JX010033	JX009866	FJ907426	JX010405	FJ917508
*C. fructicola*	ICMP 18727	*Fragaria × ananassa*	USA	JX010179	JX010035	JX009812	JX009565	JX010394	JX009682
*C. fructicola (syn. C. ignotum)*	CBS 125397(*), ICMP 18646	*Tetragastris panamensis*	Panama	JX010173	JX010032	JX009874	JX009581	JX010409	JX009674
*C. fructicola isolates*	C-557	*mango*	China	MK326868	MK473908	MK347249	MK358125	–	MK497052
*C. fructicola*	FJ28-1	*mango*	China	MH636532	MH681411	MH622474	MH622610	MH622742	–
*C. fructicola*	FJ35-5	*mango*	China	MH636538	MH681417	MH622483	MH622619	MH622748	–
*C. horii*	ICMP 10492*	*Diospyros kaki*	Japan	GQ329690	GQ329681	JX009752	JX009438	JX010450	JX009604
*C. kinghornii*	CBS 198.35*	*Phormium* sp.	UK	JQ948454	JQ948785	JQ949115	JQ949775	JQ950105	–
*C. liaoningense*	CAUOS3	*Chili pepper*	China	KP890105	KP890136	KP890128	KP890098	KP890112	KP890120
*C. nymphaeae*	CBS 129929, 2.6.23	*Fragaria ananassa*	USA	JQ948229	JQ948559	JQ948890	JQ949550	JQ949880	–
*C. nymphaeae*	CBS 126366, PD 92/785	*Fragaria ananassa*	USA	JQ948255	JQ948585	JQ948916	JQ949576	JQ949906	–
*C. nymphaeae*	CBS 515.78*	*Nymphaea alba*	Netherlands	JQ948197	JQ948527	JQ948858	JQ949518	JQ949848	–
*C. nymphaeae*	TA11	*Carya illinoinensis*	China	MH231421	MH793690	MH793689	MH891493	MH796660	MH793688
*C. orchidophilum*	CBS 632.80*	*Dendrobium* sp.	USA	JQ948151	JQ948481	JQ948812	JQ949472	JQ949802	–
*C. siamense*	ICMP 12567	*Persea americana*	Australia	JX010250	JX009940	JX009761	JX009541	JX010387	JX009697
*C. siamense*	ICMP 18121	*Dioscorea rotundata*	Nigeria	JX010245	JX009942	JX009845	JX009460	JX010402	JX009715
*C. sojae*	ATCC 62257*	*Glycine max*	USA	MG600749	MG600810	MG600860	MG600954	MG601016	–
*C. tropicicola*	MFLUCC100167	*Paphiopedilum bellatolum*	Thailand	JN050241	JN050230	–	JN050219	JN050247	–

* = Ex-type culture.

### Pathogenicity assay

Same-sized healthy lesion-free leaves were collected from ‘Mahan’ subjects growing in a disease-free orchard located in Jiande, Zhejiang Province. Fruits were collected in the same manner as the leaves a month before harvest. Pathogenicity tests were conducted on pecan following the methods described by Kanchana-udomkan et al. (2004); Than et al. (2008), and Fu et al. (2019). Healthy pecan leaves and fruits were separately surface sterilized in 1% NaClO for 5 min, washed twice with sterile-distilled water, and air dried on sterile filter paper. Each fruit was inoculated with 20 μl of a conidial suspension (1 × 10^7^ conidia/ml). The suspension was injected into the surface of non-wounded fruit using a microsyringe (Eppendorf, Shanghai, China). Control leaves and fruits were treated with 20 μl of distilled water. Each isolate was inoculated into five replicate fruits. The inoculated fruits were incubated in a moist chamber at 28°C and were examined daily for symptoms for 9 d.

### Virulence assay

Virulence assay was performed *in vivo* on seven *Colletotrichum* spp. identified as representative isolates. These were selected according to the species, orchard province, and morphological characteristics. The virulence assay was carried out in a greenhouse of the Institute of Subtropical Forestry, Chinese Academy at Forestry Sciences (Zhejiang, China) from late June to August 2020. During the 5-year black spot resistance investigation on different cultivars preserved in the Pecan Resource Garden in Jiande City, Zhejiang, China, we found highly resistant cultivars, such as ‘Kanza’, and susceptible cultivars, such as ‘Mahan’. In this experiment, annually grafted container seedlings of the highly resistant variety, ‘Kanza’, and the susceptible variety, ‘Mahan’, served as plant materials. The fourth, fifth, and sixth pairs (from bottom to top) of pinnate compound leaves were selected, surface sterilized with 75% ethanol, and rinsed with sterile water. Each leaf was impaled at its middle near the midvein and wounded five times in a 5 mm region with a sterilized needle. The 1 × 10^7^ ml^-1^ spore suspension was dropped on the wounds using a pipette. Some wounded leaves also served as controls and were inoculated with the same amount of sterile water instead of the spore suspension. Sterile damp absorbent cotton was placed on the pinnate compound leaves after air-drying, and sealed bags were used to retain the moisture. The experiment was set up with three biological replicates per isolate. The bags and cotton were removed after two days. The pathogenicity of each isolate was determined by evaluating the diameters of the disease lesions after 18 days. The one-way analysis of variance was performed with SPSS v. 16.0 software; means were compared using Duncan’s test at the significance level of 0.05.

## Results

### Fungal isolates

From 2018 to 2019, pecan leaves and shuck displaying anthracnose symptoms were collected from three pecan orchards in three provinces of China. *Colletotrichum* isolates associated with pecan anthracnose were collected from 60 affected pecan samples. Leaves were collected for fungal isolation, resulting in a total of 36 *Colletotrichum* isolates identified based on morphology and ITS sequences. A total of 11 representative isolates were chosen for further analyses based on their morphology (colony shape, color, and conidial morphology), ITS sequences, types of symptoms, and origin (**Table 1**). At least two isolates from each field were chosen for further analysis.

### Phylogenetic analyses

In total, 36 *Colletotrichum* isolates were obtained from three pecan orchards. Eleven single-spore representative isolates were used for the following morphological characterization and phylogenetic analyses (**Table 4**). These comprised two *C. fructicola* isolates from leaves and six *C. fioriniae* and three *C. fructicola* isolates from the shuck.

**Table 4:** List of 11 representative isolates of two *Colletotrichum* spp. collected from pecan in China.

Species	Isolate no.	Origin	GenBank accession number					
			*ITS*	*GAPDH*	*CHS-1*	*ACT*	*TUB2*	*CAL*
*C. fioriniae*	PCJD119	Jiande, Zhejiang	MW479426	MW634000	MW633987	MW633973	MW634014	MW633960
*C. fioriniae*	PCJD179	Jiande, Zhejiang	MW479435	MW634001	MW633988	MW633974	MW634015	MW633961
*C. fioriniae*	PCJD32	Jiande, Zhejiang	MW479436	MW634002	MW633989	MW633975	MW634016	MW633962
*C. fioriniae*	PCJD756	Jiande, Zhejiang	MW479424	MW634003	MW633990	MW633976	MW634017	MW633963
*C. fioriniae*	PCJD29	Jiande, Zhejiang	MW479427	MW634004	MW633991	MW633977	MW634018	MW633964
*C. fioriniae*	PCJD12	Jiande, Zhejiang	MW479432	MW634005	MW633992	MW633978	MW634019	MW633965
*C. fructicola*	PCYN191	Yuxi, Yunnan	MW479430	MW634009	MW633995	MW633982	MW634022	MW633969
*C. fructicola*	PCYN1751	Yuxi, Yunnan	MW479433	MW634010	MW633996	MW633983	MW634023	MW633970
*C. fructicola*	PCJD7536	Jiande, Zhejiang	MW479425	MW634011	MW633997	MW633984	MW634024	–
*C. fructicola*	PCJX073	Ji’an, Jiangxi	MW479429	MW634012	MW633998	MW633985	MW634025	MW633971
*C. fructicola*	PCJX0731	Ji’an, Jiangxi	MW479434	MW634013	MW633999	MW633986	MW634026	MW633972

Details on the origins and GenBank accession numbers are presented.

**Figure 2:**
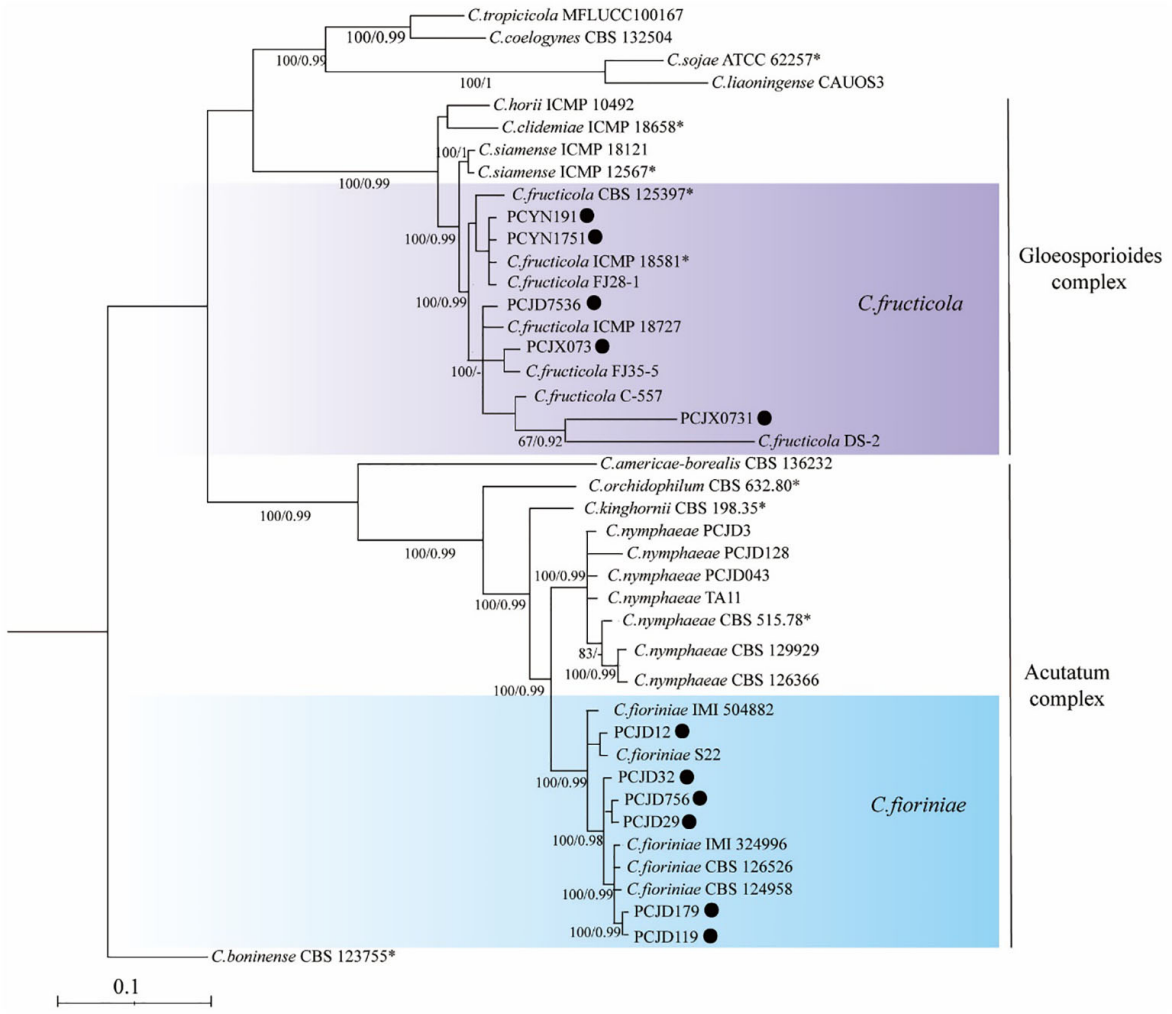
A maximum likelihood phylogenetic tree based on concatenated sequences of internal transcribed spacer (*ITS*), actin (*ACT*), calmodulin (*CAL*), glyceraldehyde-3phosphate dehydrogenase (*GAPDH*), β-tubulin (*TUB2*), and chitin synthase 1 (*CHS-1*). The tree illustrates the relationships between *Colletotrichum fioriniae* and *C. fructicola. C. boninense* (CBS 123755*) was used as an outgroup. Bayesian posterior probability values ≥ 0.90 and UFbootstrap support values ≥ 50% are shown at the nodes.

Phylogenetic analyses on the six loci (*ITS*, *ACT*, *CAL*, *GADPH*, *CHS-1*, and *TUB2*) of the *Colletotrichum* spp. included 11 isolates. The sequences of *ITS*, *GAPDH*, *ACT*, *TUB2*, *CHS-1*, and *CAL* genes of *Colletotrichum* spp. isolates from pecan were deposited to Genbank (**Table 4**). They were compared with reference sequences of *Colletotrichum* isolated from other countries and plant hosts available on GenBank **(Table 3**). The *C. boninense* (CBS 123755*) was used as the outgroup. For the maximum likelihood inference, the best fit model for the six loci was set at HKY+I+G4+F with UFbootstrap 20000. For the Bayesian inference, the best fit model was HKY+F+G4 with a gamma rate for six loci. The posterior probabilities of the Bayesian tree were consulted to confirm the topology of the maximum likelihood tree. Phylogenetic analysis provided enough information to distinguish two *Colletotrichum* species; six and five isolates belonged to *C. fioriniae* and *C. fructicola*, respectively (**Figure 2**). Among the seven isolates collected from the orchards in Jiande, six were *C. fioriniae* and one was *C. fructicola*. The four isolates from the orchards in Yunnan and Jiangxi were all *C. fructicola* (**Figure 2**).

### Morphological characteristics

**Figure 3:**
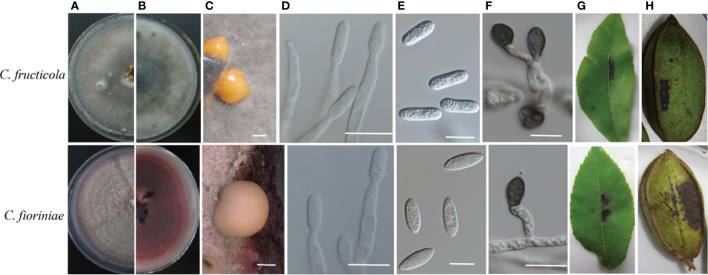
Cultural characteristics and pathogenicity of two Colletotrichum species isolated from the pecan tree. **(A, B)** front and back views of 14-days-old PDA culture; **(C)***conidiomata*; **(D)** conidiophores; **(E)** conidia; **(F)** appressoria. The symptoms were caused by **(G, H)** appearing on the leaves 6 days after inoculation and on the shuck of the fruit 20 days after inoculation with conidia. **(C, E, F)**: Bar = 10 µm. **(D)**: Bar = 50 µm.

The diameter of the colonies was measured daily for five days to calculate their mycelial growth rate (mm/d). The shape, color, and density of colonies were recorded after 14 days. Differences in colony morphology were observed between the two species identified when grown on PDA. Colonies generally showed dense, white to greyish or red growth (**Figure 3**). The mycelia of *C. fructicola* appeared dark grey on PDA plates after 14 days; their orange conidial masses were yellow and cylindrical or oval with an area of 16.8 ± 1.5 × 5.4 ± 0.4 μm^2^. *Colletotrichum fioriniae* was pinkish in color after 14 days of growth on PDA, and the conidial masses were orange. Its conidia were spindle-shaped with an area of 15.5 ± 1.5 × 5.2 ± 0.3 μm^2^. The mycelium growth rate varied from 12.3 to 15.6 mm/day (average = 14.6 mm/day) for *C. fructicola* and 10.3 to 13.2 mm/day (average = 11.24 mm/day) for *C. fioriniae*.

### Pathogenicity and virulence assays

All the *Colletotrichum* isolates were pathogenic to the detached pecan leaves and shucks. The inoculated leaves, pericarps, and nuts showed necrotic spots, while these tissues remained healthy in the controls. No lesions were induced in the control tissues that had been inoculated with sterile water. The morphology of fungal colonies re-isolated from the symptomatic shuck was the same as those produced by the original isolate used for inoculation, satisfying Koch’s postulate (**Figure 3**).

**Figure 4:**
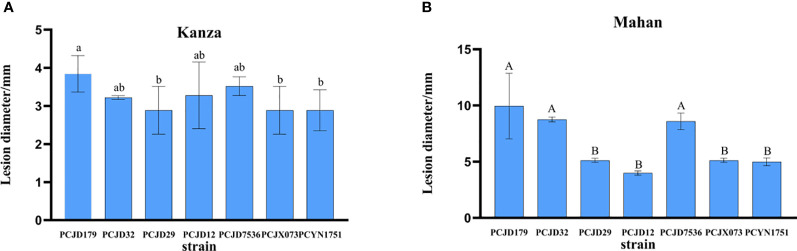
Lesion lengths on wounded pecan leaves at 18 dpi with spore suspensions of seven *Colletotrichum* spp. Different lowercase letters above the error bars indicate significant differences at *p* = 0.05. Different capital letters above the error bars indicate significant differences at *p* = 0.01. **(A)** ‘Kanza’; **(B)** ‘Mahna’.

In the virulence assay, the seven isolates caused symptoms on the leaves of 1-year-old pecan seedlings and showed different levels of virulence. The lesion diameters on the attached leaves start to differ 18 days after inoculation with various isolates (**Figures 4A, B**). No significant differences were observed in the lesion sizes produced on the highly resistant variety, ‘Kanza’, by the various isolates (*p* = 0.29), while there were significant differences on the susceptible variety, ‘Mahan’ (*p* = 0.001), indicating different virulence levels among the seven isolates. PCJD179 was noted as the strongest.

The virulence assay showed that the lesions caused by *Colletotrichum* spp. Were mainly restricted to the inoculated areas of the pecan variety, ‘Kanza’, and their diameters did not extend beyond 4 mm (**Figure 4A**). There were significant differences in the infection lesions on the ‘Mahan’ variety. The lesion diameters caused by PCJD179, PCJD32, and PCJD29 isolates of *C. fioriniae* and the PCJD7536 isolate of *C. fructicola* all exceeded 5.1 mm on ‘Mahan’. Among them, the diameter of the lesion caused by the isolate PCJD179 reached up to 10 mm, indicating that the isolate was relatively the higher virulent (**Figure 4B**).

Additionally, different isolates of *Colletotrichum* species displayed pathogenic differences. For example, the *C. fioriniae* isolates PCJD179, PCJD32, and PCJD29 produced large necrotic spots (5.1–10.0 mm). However, the spot diameters caused by PCJD12 in this species were 4.0 mm, and they were concentrated near the inoculation point. The spots produced by *C. fructicola* isolate, PCJD7536, were also significantly larger than those produced by isolates, PCJX073 and PCYN1751.

## Discussion

*Colletotrichum* spp. are important plant pathogenic fungi that cause a variety of plant diseases (Cannon et al., 2012; Dean et al., 2012; Diao et al., 2017; Guarnaccia et al., 2017). Previously, the taxonomy of *Colletotrichum* spp. has mainly been based on the host range and morphological characteristics (Von Arx, 1957; Sutton, 1980). Traditional classification methods do not effectively distinguish between the relatively complex *Colletotrichum* species because of their relatively high levels of genetic variability (Cai et al., 2009; Hyde et al., 2009; Rojas et al., 2010; Cannon et al., 2012; Damm et al., 2012a; Damm et al., 2012b). ITS sequence primers were designed to amplify the ribosomal genes of fungi. This method is a powerful means to identify and phylogenetically analyze many species at once (White et al., 1990). However, some complex species cannot be effectively identified using the ITS region alone because their support rates are low. In recent years, the identification of *Colletotrichum* species has significantly improved by combinations of multi-gene sequence and morphological analyses. Genes that produce good differentiation include glyceraldehyde 3-phosphate dehydrogenase (*GAPDH*), calmodulin (*CAL*), actin (*ACT*), β-tubulin (*TUB2*), and chitin synthase (*CHS-1*) (Damm et al., 2012a; Damm et al., 2012b; Weir et al., 2012; Damm et al., 2013; Damm et al., 2014; Yan et al., 2015; Guarnaccia et al., 2017). As a result, complexes with 15 and 14 species have been identified in the genus *Colletotrichum* (Marin-Felix et al., 2017; Damm et al., 2019). To better characterize the pathogen, multiple traits of the *Colletotrichum* spp. causing anthracnose in pecan in China are worth studying using phylogenetic analyses. Here, we detected 11 isolates from three orchards and identified them as *C. fioriniae* and *C. fructicola*. This is the first report of the two species being pathogens of pecan in China. This is also the first report of *C. fioriniae* being a pathogen of pecan worldwide. Thus, our results increase the understanding of the pathogenicity of *Colletotrichum* species against pecan.

*C. fructicola* was the first species isolated from shuck and leaves of pecan in Zhejiang, Yunnan, and Jiangxi provinces. The five isolates of *C. fructicola* mainly infected the shuck and leaves and were clustered on two branches of the phylogenetic tree. The pathogenic bacteria isolated from the diseased leaves of pecan trees from Yunnan were *C. fructicola*. The dominant species in the anthracnose of Chinese *Camellia oleifera* leaves was *C. fructicola*, which is in agreement with the observations of Li et al. (2016) and Wang et al. (2020). The six isolates of *C. fioriniae* were all isolated from the shuck and were clustered on four branches of the phylogenetic tree. This indicated that the pathogenicity of some fungi in the *Colletotrichum* genus may be tissue-specific (Fu et al., 2019; Wang et al., 2020). Moreover, anthracnose-causing *Colletotrichum* in *Camellia oleifera* Abel has previously been reported to differ in the composition, structure, and dominant phyla on leaves and fruit, where dominant phyla presented genetic differentiation among the various geographically separated populations (Li et al., 2016; Wang et al., 2020). Due to the small sample size, our study failed to reflect the distribution of *Colletotrichum* species and genetic variation in the mechanisms by which they cause anthracnose in pecan trees. This information could provide a theoretical basis for formulating targeted disease prevention and control strategies.

*C. fructicola* can cause anthracnose in the plants of several genera, including *Citrus reticulata*, *Capsicum annuum*, *Camellia sinensis*, and *Mangifera indica* (Huang et al., 2013; Lima et al., 2013; Liu et al., 2015; Diao et al., 2017). This *Colletotrichum* species has been associated with a certain geographical preference and is mainly distributed in the Yangtze River Basin in China (Fu et al., 2019). We found that *C. fructicola* was isolated from Zhejiang, Jiangxi, and Yunnan provinces. Additionally, the isolates from these three locations were clustered on two branches of the phylogenetic tree. The PCJD7536, PCJX073, and PCJX0731 isolates isolated from the shuck were clustered on one branch, and the PCYN1751 and PCYN191 isolates from the leaves were clustered on one branch. The pecans in Jiangxi Province were introduced from Jiande, Zhejiang Province. PCJD7536, PCJX073, and PCJX0731 were clustered in one branch, which might be related to the plants’ origin or the host tissues (Wang et al., 2020; Fu et al., 2019). In China, the cultivation of pecan was initiated in Yunnan and Zhejiang in the 1960s. Because of the recent initiation of large cultivation of pecan in Jiangxi, most of the pecan here was cultivated from Zhejiang-primed cultivars. Thus, the introduced species were genetically similar. In Yunnan, pecan grows as a native variety. The local climate and ecological environment differ from Zhejiang and are geographically distant. A larger number of samples are thus needed for further in-depth investigations.

Differences were revealed in the pathogenicity of the species or isolates of *Colletotrichum*. *C. fioriniae* and *C. fructicola* cause disease on the leaves and shuck of pecan trees, while the seven isolates of both species presented varying degrees of pathogenicity. Pathogenicity differentiation between and within the *Colletotrichum* spp. has been previously reported in chili (*Capsicum* spp.), citrus (*Citrus* spp.), and pear (*Pyrus* spp). Pathogenicity associated with various regions, varieties, and tissues of the host is also established (Diao et al., 2017; Guarnaccia et al., 2017; Fu et al., 2019). The present study preliminarily determined the pathogenicity of *Colletotrichum* which inflicts pecan trees with anthracnose. The symptoms of anthracnose are affected by various factors, including temperature, relative humidity, variety, and the number of pathogens (Han et al., 2016; Mo et al., 2018). There may be some differences between the results of virulence assays performed in the laboratory and those on trees growing in natural environmental conditions in the orchards.

## Data availability statement

The dataets presented in this study can be found in online repositories. The names of the repository/repositories and accession number(s) can be found in the article/Supplementary Material.

## Author contributions

JC conceived this project. JC and YZ designed experiments and interpreted the results. JC wrote the manuscript. FZ and JS provided technical guidance for the experiment. JC, DW, and YZ performed the experiments and analyzed the data. XY provided experimental materials and funds. All authors contributed to the article and approved the submitted version.

## Funding

The work was supported by the Fundamental Research Funds for the Central Non-Profit Research Institution of CAF (CAFYBB2020SY014).

## Conflict of interest

The authors declare that the research was conducted in the absence of any commercial or financial relationships that could be construed as a potential conflict of interest.

## Publisher’s note

All claims expressed in this article are solely those of the authors and do not necessarily represent those of their affiliated organizations, or those of the publisher, the editors and the reviewers. Any product that may be evaluated in this article, or claim that may be made by its manufacturer, is not guaranteed or endorsed by the publisher.
